# Insight into the Ex Situ Catalytic Pyrolysis of Biomass over Char Supported Metals Catalyst: Syngas Production and Tar Decomposition

**DOI:** 10.3390/nano10071397

**Published:** 2020-07-18

**Authors:** Mian Hu, Baihui Cui, Bo Xiao, Shiyi Luo, Dabin Guo

**Affiliations:** 1 College of Environment, Zhejiang University of Technology, Hangzhou 310032, China; mianhu@zjut.edu.cn; 2 School of Environmental Science & Engineering, Huazhong University of Science and Technology, Wuhan 430074, China; xiaobo1958@126.com; 3 Institute of Hydrobiology, Chinese Academy of Sciences, Wuhan 430072, China; cuibaihui@ihb.ac.cn; 4 State Key Laboratory of Physical Chemistry of Solid Surfaces, College of Chemistry and Chemical Engineering, Xiamen University, Xiamen 361005, China; luoshiyi@xmu.edu.cn

**Keywords:** ex situ, catalytic pyrolysis, biomass, syngas production, tar decomposition

## Abstract

Ex situ catalytic pyrolysis of biomass using char-supported nanoparticles metals (Fe and Ni) catalyst for syngas production and tar decomposition was investigated. The characterizations of fresh Fe-Ni/char catalysts were determined by TGA, SEM–EDS, Brunauer–Emmett–Teller (BET), and XPS. The results indicated that nanoparticles metal substances (Fe and Ni) successfully impregnated into the char support and increased the thermal stability of Fe-Ni/char. Fe-Ni/char catalyst exhibited relatively superior catalytic performance, where the syngas yield and the molar ratio of H2/CO were 0.91 Nm3/kg biomass and 1.64, respectively. Moreover, the lowest tar yield (43.21 g/kg biomass) and the highest tar catalytic conversion efficiency (84.97 wt.%) were also obtained under the condition of Ni/char. Ultimate analysis and GC–MS were employed to analyze the characterization of tar, and the results indicated that the percentage of aromatic hydrocarbons appreciably increased with the significantly decrease in oxygenated compounds and nitrogenous compounds, especially in Fe-Ni/char catalyst, when compared with no catalyst pyrolysis. After catalytic pyrolysis, XPS was employed to investigate the surface valence states of the characteristic elements in the catalysts. The results indicated that the metallic oxides (Me_x_O_y_) were reduced to metallic Me^0^ as active sites for tar catalytic pyrolysis. The main reactions pathway involved during ex situ catalytic pyrolysis of biomass based on char-supported catalyst was proposed. These findings indicate that char has the potential to be used as an efficient and low-cost catalyst toward biomass pyrolysis for syngas production and tar decomposition.

## 1. Introduction

In recent years, biomass has attracted much attention mainly because it can be used as a renewable resource to produce various high-quality chemicals, multi-functional char-based materials, and fuel products, which can not only reduce the burden of relying on fossil resources, but also are regarded as a meaningful way toward CO_2_ emissions [1,2,3,4,5,6]. Currently, biomass energy accounts for about 9% of the world’s total energy supply that plays a key role in tacking issues of energy-supply security and environmental problems [7]. Some promising thermochemical conversion processes, such as pyrolysis, gasification, hydrothermal liquefaction etc., have been explored toward enhanced energy and resource recovery from biomass [8,9,10,11]. Among them, pyrolysis is recognized as an advanced and practical technology for high-value utilization of the biomass [12,13]. Generally, pyrolysis of organic substances produces three phases of matter. It produces gaseous products, including syngas, methane, short hydrocarbon chain gases, and carbon dioxide. It also produces liquid and carbon products [14]. However, during the pyrolysis process, some unpleasant by-products (e.g., tar, NO_x_, and ash) are inevitably produced. Tar, which generally includes aromatic compounds with one-ring to five-rings, oxygen-containing hydrocarbons, and polycyclic aromatic hydrocarbons, is one of the main problems hindering the industrialization of pyrolysis technology, because it condenses easily [15,16,17].

Catalytic pyrolysis is an effective route to improve tar quality, which makes pyrolysis volatiles occur in a series of secondary reactions, and make the heavier component split into light oil and gas, as well as transform the oxygen in tar to CO_2_, CO, and H_2_O through decarboxylation, decarbonylation, and dehydration reactions, respectively [18,19]. In addition, the introduction of a catalyst in the pyrolysis process can reduce the activation energy of the reaction, thereby greatly reducing the required pyrolysis temperature [20,21]. Generally, the catalytic pyrolysis can be classified into in situ and ex situ configurations according to the location of the catalyst. In situ catalytic pyrolysis means that the catalyst and feedstock are mixed together, and the pyrolysis and vapor catalytic reforming/cracking processes take place in the same reactor; therefore, the capital and operating costs are reduced. Ex situ catalytic pyrolysis means that the catalyst and feedstock are loaded separately in different reactors. The pyrolysis vapor flows out from the pyrolysis reactor and introduces into the catalytic reactor for catalytic reforming/cracking [22,23]. Compared with the in situ catalytic pyrolysis, the ex situ catalytic pyrolysis can adjust the temperature of the pyrolysis reactor and catalytic reactor, respectively, making the reaction system more controllable and flexible. Meanwhile, the probability of contact between pyrolysis vapors and catalyst is higher in an ex situ catalytic pyrolysis system. In addition, compared with in situ catalytic pyrolysis, the post-reaction catalyst can be easily separated for next cycle [24]. Various char-based catalysts were developed by Han et al., [25] for the ex situ catalytic upgrading of coal pyrolysis tar and the results exhibited that the catalytic upgrading resulted in a lower total yield of tar and a higher yield of non-condensable gas. Gamliel et al. [26] performed a comparison between in situ and ex situ catalytic fast pyrolysis, and the results indicated that the ex situ catalytic fast pyrolysis produced more gases yield and aromatics in the bio-oil than in situ catalytic fast pyrolysis. Depending on the above statement, thus, in this study, the ex situ catalytic pyrolysis system was self-designed for investigation of biomass pyrolysis.

Various catalysts, such as nickel-based catalysts [27], noble-metal-based catalysts [28], transition metal catalysts [29], alkali metal catalysts [30], natural catalysts [31], zeolite catalysts [32], and carbon-supported catalysts [33] have been investigated for syngas production and tar decomposition from biomass. In general, Ni-based catalysts are considered to be better for catalytic cracking/reforming of tar. Ni metal catalysts can not only activate hydrogen, but also significantly inhibit the polymerization of unsaturated hydrocarbons that lead to coke formation. However, compared with Ni-based catalysts, Fe-based catalysts are much cheaper, more abundant, and more environmentally friendly. Moreover, iron oxides have a variety of different physicochemical properties, such as Fe_2_O_3_ and Fe_3_O_4_, which may increase catalytic activity or reduce coking deposition. Moreover, the mono- or bi-metallic catalysts, such as Fe-Ni/HZSM-5 [34], Co-Fe/Al_2_O_3_ [35], and Ni/char [8] are beneficial for tar decomposition. Catalyst supports are usually supported by metal oxides or natural minerals, and these supports are relatively expensive. The nature of a catalyst support plays an important role in the reactivity of the catalyst. Char, as a low-cost porous pyrolysis residue, is considered to be an effective catalyst/support in tar removal, due to its inherent catalytic alkali and alkaline earth metallic species, as well as the defected carbon structural units in chars [36,37]. Meanwhile, the porous structure of char facilitates metal impregnation and makes tar more accessible to catalytic active sites. Hence, char, which could easily be obtained from solid waste pyrolysis, may have the potential to upgrade the bio-oil quality and syngas yield. However, although this cheap and green char catalyst has received more attention on catalytic pyrolysis of biomass, it is still worth it to understand the catalytic activities and mechanisms of char or char-supported catalysts on tar decomposition and syngas production.

In this work, ex situ catalytic pyrolysis of biomass over char-supported Ni and/or Fe catalysts for syngas production and tar decomposition was investigated. The objectives of this this study were to (1) synthesize char-based catalysts by impregnation with metal (Fe and/or Ni), (2) compare the catalytic activity of char-based catalysts for syngas production and tar decomposition, and (3) discuss the catalytic mechanism of biomass over char-based catalyst.

## 2. Materials and Methods

### 2.1. Pyrolysis Feedstock

Pine sawdust (PS) used in this study was collected from a factory in Wuhan, China. Sun-drying for 7 days was performed for PS, to reduce the moisture content; then it was crushed and sieved to obtain a particle size <0.107 mm before using. Detailed information about the sample is listed in Table 1. Fe(NO_3_)_3_·9H_2_O and Ni(NO_3_)_2_·6H_2_O chemicals employed for the catalyst synthesized were analytical reagent grade.

### 2.2. Catalysts Preparation

Char, as a catalyst or catalyst support, was derived from a biomass steam gasification system, and its main physicochemical properties are also shown in Table 1. Carbon content of char is 76.12 wt.% with limited oxygen (8.12 wt.%). The Brunauer–Emmett–Teller (BET) specific surface area is 252.13 (m^2^/g), which should make it good as a catalyst support. Before being used as a catalyst support, char needs to be sieved (0.5–1 mm), washed, and dried (80 °C). Char-supported iron and nickel catalysts were prepared by impregnation method. Firstly, 44.0 g of char support was impregnated by using a mixed aqueous solution of Fe(NO_3_)_3_·9H_2_O (21.6 g) and Ni(NO_3_)_2_·6H_2_O (14.8 g), and stirred at room temperature for 12 h. After that, the sample was dried at 105 °C for 24 h. The prepared nanoparticles catalyst was marked as Fe-Ni/char catalyst. Meanwhile, char without metals was used as the baseline for comparison.

### 2.3. Apparatus and Procedure

**Figure 1:**
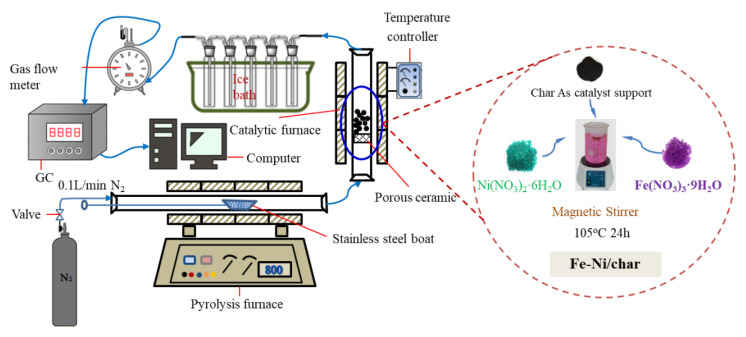
Schematic diagram of ex situ catalytic pyrolysis system.

In this study, the catalytic pyrolysis of different types of char and Ni-6/char was used as the catalyst. Ex situ catalytic pyrolysis of biomass was conducted in a self-built equipment, as shown in Figure 1. The reaction system consists of carrier gas unit, pyrolysis furnace, catalytic furnace, condenser, gas flow meter, and GC for gas analysis. Two quartz glass tubes (i.d. = 75 and 80 mm, effective length = 700 and 800 mm, respectively) were used as the pyrolysis and catalytic bed reactor, respectively.

The 5.0 g catalyst was preloaded into the catalytic bed, followed by protection by 0.1 L/min of N_2_ for 20 min. The temperatures of the pyrolysis and catalytic reactors were both controlled at 800 °C. We loaded 5.0 g of PS onto a heat-resistant boat and pushed it into the middle of the pyrolysis reactor, when the desired temperature stabilized. The pyrolysis gas passed through a condenser, and a mixture liquid (tar and water) was obtained.

Flow meter was employed to determine the volume of non-condensable syngas, and its composition was measured by a Gas Chromatography (GC).

After each experiment, the liquid products which came from the ice bath and connection tubes were collected. According to the standard ASTM D244 and IP 291.1 methods, water and tar were further separated. The mass of tar was weighted and recorded as the tar yield.

### 2.4. Method of Sampling and Analysis

The syngas was analyzed by using GC 9800T with a thermal conductivity detector. The column used was TDX-01 for the analysis of H_2_, CO, CO_2_, and CH_4_. The temperatures 200, 85, and 90 °C were employed, respectively, for the injector, oven, and detector. The argon was applied for carrier gas, while the standard gas mixtures were used for quantitative calibration.

BET specific surface area (S*_BET_*) was determined by the Brunauer–Emmett–Teller (BET) equation. The total pore volume (V*_total_*) was investigated by single point adsorption total pore volume analysis. Average pore diameter (D) was obtained by 4V/S*_BET_* based on BET method. The thermal stability of fresh catalysts was characterized by the TG/DTA Synchronous analyzer (Diamond TG/DTA, PerkinElmer Instruments, Norwalk, CT, USA). Scanning electron microscopy (SEM) coupled with energy-dispersive X-ray fluorescence spectroscopy (EDX) with a Quanta 200 (FEI Company, Eindhoven, Nederland) was employed to examine the structural characteristics and elements distribution of catalysts. Before and after catalytic reforming, X-ray photoelectron spectroscopy (XPS, Shimadzu- Kratos GROUP PLC), using Al Kα as an X-ray source, was applied to analyze the species of the elements. C 1s set as 285.0 eV was used as an internal standard.

Tar samples were analyzed by using an Agilent GC/MS (7890A/5975C, Agilent, Santa Clara, CA, USA) with a capillary column (HP-5MS) (30 m × 0.25 mm × 0.25 mm) to quantify the relatively light components of the tar. The identification of the peaks in the chromatogram were determined by using NIST98 mass library.

## 3. Results and Discussion

### 3.1. Characterization of Fresh Catalysts

**Figure 2:**
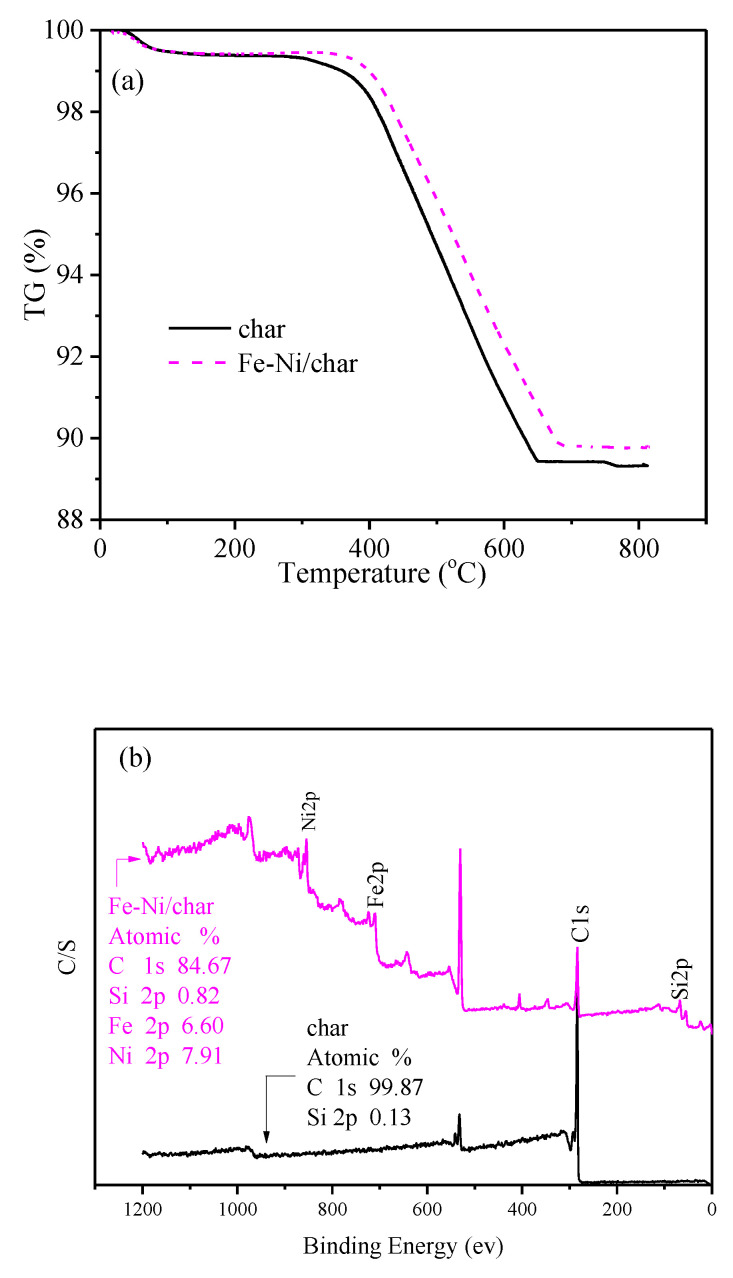
TG curves (**a**) and XPS (**b**) analysis of different fresh catalysts.

Thermogravimetric analysis (TGA) was used to determine the thermal stability of fresh char and char-supported catalysts at a heating rate of 15 °C/min and N_2_ flow rate of 100 mL/min, and the results are presented in Figure 2a. It can be seen that, when the temperature was above about 350 °C, the weight of char/char-supported catalysts decreased rapidly. Thermal decomposition resulted in the greatest mass loss in the temperature range of about 350–663 °C for char, which differed with Fe-Ni/char. The mass remaining, compared to the initial mass, is 89.42% for char, and 89.81% for Fe-Ni/char, which indicated that the order of thermal stability of char and char supported catalysts is Fe-Ni/char > char.

The SEM images of the fresh char and Fe-Ni/char catalysts with elements distribution by EDS analysis are shown in Supplementary Materials Figure S1. From the image in Supplementary Materials Figure S1a, it can be seen that the morphological characterizations of rough, corrugated, and many cavities are observed on the char samples, which can be proved by the result of specific surface areas (252.13 m^2^/g). Meanwhile, the EDS result of char indicated that some inorganic elements (such as silicon and potassium) existed on the surface of char. After Ni and Fe loading, small particles can be observed, and their specific surface areas accordingly decreased (74.89 m^2^/g for Fe-Ni/char), as shown in Table 2. Additionally, the EDS results of the Fe-Ni/char catalyst are presented in Supplementary Materials Figure S1b and indicate that the metallic matters were successfully inserted into the char framework.

The nitrogen adsorption–desorption isotherms and pore size distribution of different fresh catalysts were analyzed, and the results are shown in Supplementary Materials Figure S2. By analyzing the pore diameter distribution of char, it can be seen that the influence of loading Fe and Ni on char structure reduces the average pore diameter of char. According to the Brunauer–Deming–Deming–Teller (BDDT) classification, the isotherms of char and Fe-Ni/char could be type IV, due to to the porous walls covering the surface followed by pore filling associated with various hysteresis loops. The above results shown that char-based catalysts exhibited good catalytic activity during the catalytic pyrolysis process.

XPS analysis of catalysts was performed, and the results are shown in Figure 2b. The surface of the char was enriched with only carbon and oxygen (not given but really exist in the binding energy of about 529 eV) and a small amount of silicon, while Fe and Ni appear on the corresponding char-supported metal catalysts after impregnation. These results are consistent with the consequences of EDS analyses, which indicated that the Fe and Ni were successfully impregnated into the char support.

### 3.2. Syngas Yield and Composition

As a key precursor of fuel products, syngas has different applications, depending on the ratio of H_2_/CO. When using dimethyl ether as the target product, the ratio is 1, while the synthesis of methanol and long-chain alkanes requires a ratio of 2 or higher [38]. However, the ratio of the H_2_/CO in syngas is generally kept at a lower level, especially when air and oxygen are used as gasifying agents [39]. Biagini et al. [40] investigated the gasification of agricultural residues, and the results indicated that the H_2_/CO ratio of the syngas was lower than 1. Weiland et al. [41] gasified the wood powder with oxygen, and the ratio of H_2_/CO was between 0.54 and 0.57. Therefore, catalytic pyrolysis/gasification should be performed to increase the syngas H_2_/CO ratio.

**Figure 3:**
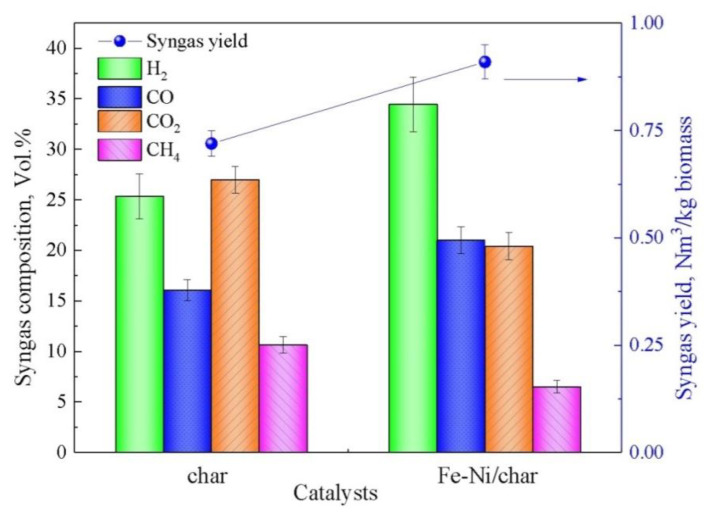
Syngas yield and composition with different catalysts.

The syngas yield and composition from biomass catalytic pyrolysis processes under different employed catalysts are shown in Figure 3. Compared with the char as catalyst, the syngas yield is increased in the presence of the Fe-Ni/char catalyst. The increase in syngas production is mainly due to the complex thermochemical reactions between three phase products (gas, char, and tar) and the catalyst. More specifically, the syngas yield (0.91 Nm^3^/kg biomass) under the Fe-Ni/char catalyst is higher than in the case of the char catalyst, indicating that its catalytic performance for syngas production is better. Figure 3 also shown the content of the four main gas components, namely H_2_, CO, CO_2_, and CH_4_, under different catalysts. In comparison with the char as catalyst, the volume concentrations of H_2_ and CO increased, whereas the CO_2_ and CH_4_ volume concentrations decreased in the presence of the Fe-Ni/char catalyst. The char-supported Fe and Ni catalyst exhibited much higher catalytic activity than char catalyst, which in turn promoted tar catalytic cracking and syngas generation. Meanwhile, under the different catalysts’ conditions, the molar ratio of H_2_/CO was 1.57 and 1.64, respectively, for char and Fe-Ni/char. Therefore, the syngas obtained from these char and Fe-Ni/char catalysts can act as feedstock for Fischer–Tropsch synthesis for the production transportation fuels [42].

### 3.3. Tar Yield, Conversion Efficiency, and Composition Analysis

**Figure 4:**
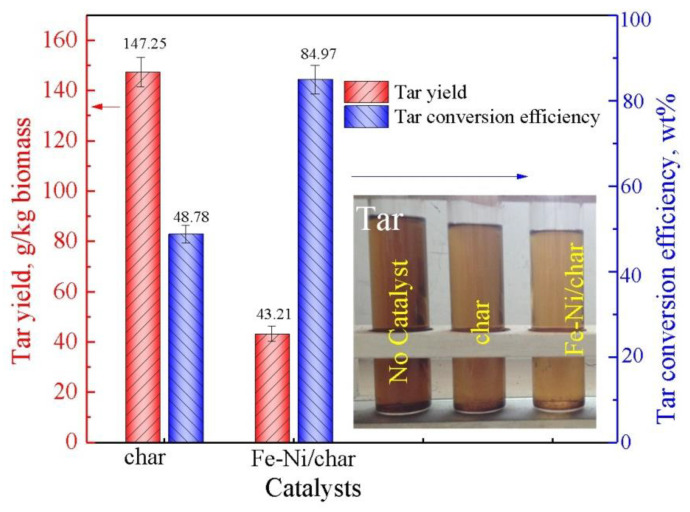
Tar yield and conversion efficiency.

The results of tar yield and conversion efficiency under different char-based catalysts by ex situ catalytic pyrolysis are described in Figure 4. In the absence of a catalyst, the tar yield is 287.5 g/kg biomass, while with char as a catalyst in the catalytic pyrolysis process, the tar yield is 147.25 g/kg biomass. The tar conversion efficiency is 48.78%, indicating that the char has fair catalytic activity as a catalyst, which is due to its inherent catalytic alkali and alkaline earth metallic, as well as the defected carbon structural units in chars [18]. Moreover, it can be seen intuitively that the Fe-Ni/char catalyst has significantly higher catalytic activity than the char catalyst, and the tar conversion efficiency is significantly increased (84.97 wt.%), which indicates that the Fe-Ni/char catalyst indeed has higher tar catalytic decomposition performances. The results indicated that the gas yields of char and Fe-Ni/char as catalysts at 800 °C were 0.72 and 0.91 Nm^3^/kg, tar yields were 147.25 and 43.21 g/kg, and char yields were 220 and 130 g/kg, respectively. In the previous studies, Shen et al. [43] used rice-husk-char-supported nickel–iron catalysts for catalytic cracking tar, and the results showed that the tar conversion efficiency was the best (93%) when the Ni-Char was used as catalyst. In their studies, the monometallic catalysts and bimetallic catalysts also presented high tar conversion efficiency (>80%), indicating that char could be used as a green solid resource in applications for energy recovery.

In addition, under the ex situ conditions, the volatiles from the primary pyrolysis furnace enters the catalyst furnace and reaction with the catalyst, which further decomposes the oxygen-containing functional groups in the volatiles, and the acid sites in the catalyst surface can promote various secondary reactions releases of H_2_O and CO_2_, reduce the tar content, and increase the amount of small hydrocarbon compounds.

The ultimate analysis results of tar under different catalyst conditions are shown in Table 3. The two main elements in tar are C and O, followed by H and N, and only a small amount of S (0.023–0.048 wt%) has been detected. This result indicates that the concern about SO_x_ emissions or corrosion during tar use is negligible. Assuming that the S element is ignored, the molecular formula of tar can be written as CH_x_O_y_N_z_, based on a C atom, and the results are also presented in Table 3. It can be seen that, after catalytic pyrolysis, the content of C increased dramatically, and the content of N and O decreased significantly, while the content of H and S changed slightly. The C content in the catalyst increased from 54.67 to 77.43 wt.%, the O content was reduced from 31.863 to11.856 wt.%, and the O/C molar ratio was significantly reduced from 0.43 (without catalyst) to 0.115 (Fe-Ni/char). The decreased in O/C molar ratio indicated that the O-containing compounds in tar decreased after catalytic pyrolysis. Meanwhile, compared with the case of no catalyst, the molar ratio of H/C in its produced tar decreased for all the tested catalysts. As shown in Figure 3, the yield of syngas increases after catalytic pyrolysis, which reduced the H/C molar ratio and derived the tar decomposition. Moreover, the N contents in these tars are high, especially in the condition of no catalyst (6.2 wt.%), indicating that there is a potential risk of polluting by the NO_x_ when utilizing these tars. After catalytic pyrolysis, the N content decreased in each catalyst, possibly due to the conversion of N-containing compounds to NO_x_. Actually, mineral matters, such as potassium, calcium, iron, aluminum, etc., in biomass/char have great effects on N-conversion to NO_x_ [44]. Yi et al. [45] investigated the influence of mixed-Fe/Ca additives on nitrogen transformation during protein and amino acids pyrolysis, and the results indicated that the calcium can promote NH_3_ formation and iron increases HCN yield. The synergistic effects of mixed-Fe/Ca additives enhanced the removal of HCN and NH_3_. Meanwhile, Meesuk et al. [46] studied the catalytic reforming of nitrogen-containing volatiles evolved through pyrolysis of composed pig manure and revealed that Ni-based catalysts have high activity for conversion of nitrogen species in volatiles into N_2_.

Supplementary Materials Table S1 shows the GC–MS analyses’ results of main compounds identified in the tar. It should be noted that only the matched-degrees ≥ 90% are listed in the results. About 100 compounds are tentatively identified, which can be divided into three groups: aromatic hydrocarbons, oxygenated compounds, and nitrogenous compounds.

It can be seen that catalytic pyrolysis with the char and Fe-Ni/char catalysts generated a higher percentage of aromatic hydrocarbons, compared with no catalyst pyrolysis, indicating that employed catalyst increases the percentage of aromatic hydrocarbons composition in the tar. Vichaphund et al. [47] also confirmed that the content of aromatic hydrocarbons in tar from Jatropha residues was increased under the condition of fast catalytic pyrolysis. The formation of aromatic hydrocarbons could be beneficial to improve calorific values of tar. In this study, aromatic hydrocarbons are composed of monocyclic aromatic hydrocarbons (MAHs) and polycyclic aromatic hydrocarbons (PAHs), where PAHs could be divided into light polyaromatic hydrocarbons (LPAHs) (two to three rings) and heavy polyaromatic hydrocarbons (HPAHs) (larger than three rings). As shown in Supplementary Materials Table S1, under the no-catalyst condition, the main components’ MAHs in tar include benzene derivatives, while LPAHs include naphthalene derivatives, fluorene, and anthracene derivatives; and HPAHs include fluoranthene, pyrene-1-methyl, and triphenylene. The total content of HPAHs is 0.6828%. After catalytic pyrolysis, the total content of HPAHs is as the order of 0.5408% (char as catalyst) > 0.4089% (Fe-Ni/char as catalyst). It is obvious that HPAHs can condense at a high temperature with low concentration. After catalytic pyrolysis, the content of HPAHs in the tar is continuously reduced, which is beneficial to the subsequent treatment, making it more easy and efficient. In addition, it is also found that the molecular weight of tar decreased, which is consistent with the conclusions of ultimate analyses (Table 3). Obviously, the total contents of MAHs are higher than that of PAHs under all experimental conditions. The reason should be due to the formation of PAHs mainly derived from a series of reactions between MAHs and another oxygenate through the loss of H_2_O and CO. Furthermore, PAHs are easily deposited on the surface of the catalyst to form coke.

In this study, the main oxygen-containing compounds in tar included aldehydes, ketones, phenols, ethers, and esters. The incontrovertible fact is that the presence of aldehydes and ketones in the tar results in instability, while ethers and ester can reduce the calorific value of the tar. It can be seen from Supplementary Materials Table S1 that, when the catalyst is used, the percentage of oxygenated compounds shows a downward trend, indicating the occurrence of deoxygenation reactions (remove oxygen as CO, CO_2_, and H_2_O). For example, ketones might transform into aromatics and H_2_O through condensation and decomposition reactions, while aldehydes might occur through dehydration reaction, and ester might occur through decarboxylation reactions with char-based catalysts. Especially, when Fe-Ni/char is used as a catalyst, the percentage of oxygenated compounds is only 7.9777%, which shows a significant decline compared with that in the no-catalyst pyrolysis (17.2932%). This is mainly due to the fact that the Ni can enhance the cracking of O–H, C–H, and C–C and have high selectivity for hydrodeoxygenation of various C–O and C=O bonds during the tar upgrading process [48]. Compared with no catalyst pyrolysis, catalytic pyrolysis mainly generated ethyl acetate, while no methyl propionate was generated. This could be due to the methyl propionate being converted to ethyl acetate by the isomerization reaction. Meanwhile, the percentages of phenolics in tars after catalytic pyrolysis were all decreased, indicating that the hydrocarbons might have been produced from phenolics.

The nitrogenous compounds in this study mainly consist of pyrimidine, pyrazine, pyridine, pyrrole, quinolone, and nitrile, which, due to the occurrence forms of nitrogen in biomass, mainly exist in the protein, accounting for 80–85% of total nitrogen in biomass [44]. Nitrogenous compounds in tar may cause potential NO_x_ pollution during combustion. As can be seen from Supplementary Materials Table S1, after catalytic pyrolysis, all the percentages of nitrogenous compounds are decreased significantly, especially under the Fe-Ni/char catalysts’ conditions, indicating that the char-supported Fe-Ni catalyst can improve the quality of the tar. Meanwhile, compared with the char as catalyst, the percentage of nitrogenous compounds is 1.9657% when Fe-Ni/char is used as the catalyst, which less than 2.6707%. This result indicates the Fe-Ni-metals-loaded char catalyst facilitates thermal cracking of nitrogenous compounds with the release of gas-N [45].

In conclusion, the improvement of tar quality in this study was achieved through ex situ catalytic pyrolysis of biomass on a char-based catalyst. At the same time, with the decrease of the content of oxygen-containing compounds and nitrogen-containing compounds, the content of aromatic hydrocarbons is higher, indicating that the char-supported Ni and Fe catalysts are beneficial to improving the quality of pyrolytic tar.

### 3.4. XPS Analyses of Catalyst

**Figure 5:**
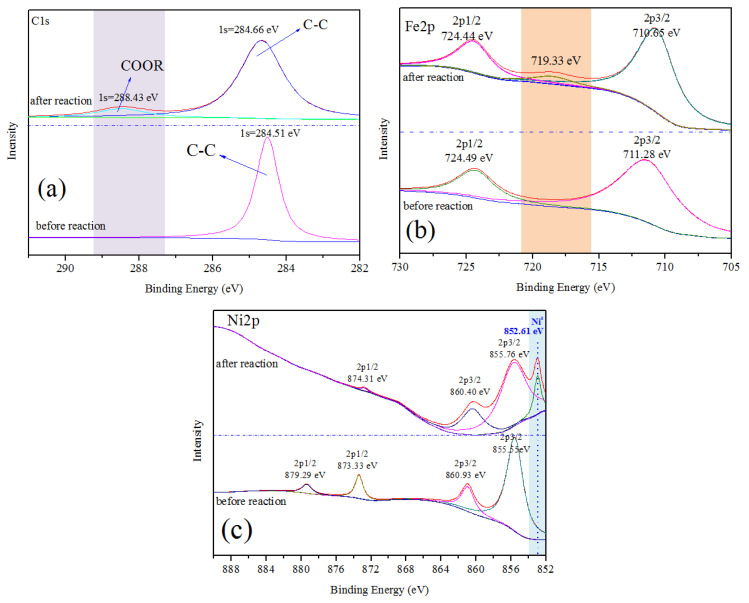
XPS patterns of Fe-Ni/char catalyst before and after reaction. (**a**) C1s spectra; (**b**) Fe2p spectra; (**c**) Ni2p spectra.

In order to investigate the surface valence states of the characteristic elements in the catalysts before and after reactions, XPS measurement is employed. The high-resolution XPS scans of the C 1s, Fe 2p and Ni 2p regions for the representative catalyst Fe-Ni/char are depicted in Figure 5. The C 1s spectrum of fresh Fe-Ni/char catalyst is deconvoluted into the carbon atom in the form of sp^2^ hybridized graphitized carbon (C–C) (Figure 5a). The peak at the binding energy of about 284.5 eV indicated that the most carbons in the char-supported catalyst are aromatic carbons. Garcia-Bordeje et al., [49] studied the hydrothermal carbonization of cellulose and found that a prominent signal of sp^2^ carbons in aromatic rings, consistent with the observation in this work. After catalytic pyrolysis, a peak at the binding energy of 288.43 eV of C 1s appeared which correspond to carboxyl group (COOR). The reason of this changed might be attributed to the tar is decomposed to form coke and deposited on the surface of char.

The Fe 2p spectrum of before and after reaction Fe-Ni/char comprises two and three peaks with differentiated binding energy values, respectively (see Figure 5b). Usually, the peak position of Fe 2p1/2 and Fe 2p3/2 determines the ionic states of Fe. Meanwhile, the satellite peak positions for the Fe 2p1/2 and Fe 2p3/2 peaks are very sensitive to the oxidation states. Therefore, these peaks could be used for determining the ionic states of iron. Before catalytic pyrolysis, the Fe 2p3/2 with binding energy of 711.28 eV identified as the peak of FeO(OH) or other substances between Fe_2_O_3_ and FeO(OH), while the Fe 2p1/2 peak at a binding energy of 724.49 eV is ascribed to Fe^3+^ cations. After catalytic pyrolysis, a peak of Fe 2p3/2 with binding energy of 710.65 eV indicated that both Fe^2+^ and Fe^3+^ should exist. The shifts from 711.28 to 710.65 eV indicate a reduction of Fe^3+^ to Fe^2+^ during the catalytic pyrolysis process. Noteworthy, after catalytic pyrolysis, a peak at binding energy of 719.33 eV appears which associated with Fe^0^. This result manifests that there were oxidation-reduction reactions during the catalytic pyrolysis process. The iron oxide could be reduced by carbon and/or reducing gas (H_2_, CO), which comes from biomass pyrolysis.

In the Ni 2p spectrum (Figure 5c), before catalytic pyrolysis, there are four peaks with the binding energy in the range of about 855–879 eV. The Ni 2p3/2 with binding energy of 855.55 eV identified as the peak of Ni(OH)_2_, while the Ni 2p1/2 with the binding energy of 873.33 and 879.29 eV could be assigned to Ni^2+^ on the surface of catalyst. Usually, the binding energy of NiO usually distributes at 853.3 eV in the standard spectrum. However, the Ni 2p3/2 peak of before and after reaction catalysts centered at 855.55 and 855.76 eV, respectively, which can be attributed to the interaction with the catalyst support indicating the presence of NiO or other nickel compounds. After catalytic pyrolysis, a special peak is observed at binding energy of 852.61 eV, which can be attributed to metal Ni. Carbothermal and hydrogenation reductions of Ni^2+^ to Ni should be the main reasons.

### 3.5. Tar Catalytic Conversion Mechanisms

**Figure 6:**
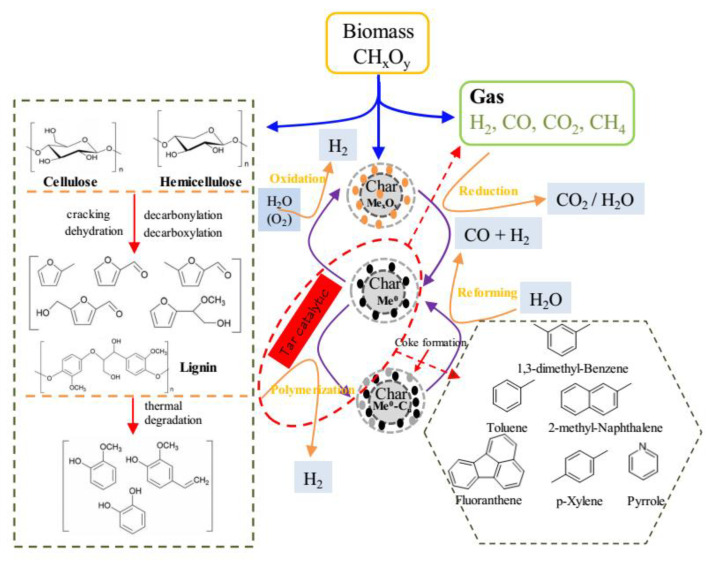
Proposed reaction pathway for ex situ catalytic pyrolysis of biomass.

In order to more clearly understand the catalytic pyrolysis process of the tar, based on the above discussion, the main reactions pathway involved during ex situ catalytic pyrolysis of biomass is summarized in Figure 6. For ex situ catalytic pyrolysis process, biomass pyrolysis is the first step. Cellulose, hemicellulose, and lignin are the three main components in biomass. Cellulose, as the main component of lignocellulosic biomass, can form furan compounds through a series of dehydration, cracking, decarboxylation, and decarboxylation reactions during thermal degradation. As such, it is evidenced that hemicellulose was likely to be depolymerized into furan compounds, which is comparable with the result of cellulose degradation. Unlike cellulose and hemicellulose, lignin is primarily decomposed into phenolic compounds. During the above pyrolysis process, non-condensable gas and a solid residue called char are also produced.

The pyrolysis vapors then pass through the catalyst bed and undergo the catalyst pyrolysis upgrading. In this study, char-supported Fe/Ni catalysts were employed. According to the XPS analysis results, Fe and Ni are existed in the form of oxidation state in fresh catalysts (Me_x_O_y_ refers to metallic oxides, and M refers to Fe, Ni). When the non-condensable gases which contain reducing gas (H_2_, CO) pass through the catalyst bed, the Me_x_O_y_ is reduced to Me^0^. Meanwhile, the condensable gases (refer to tar) are adsorbed on the surface of char-supported catalysts. The reduced metal Me^0^ active sites at the catalysts’ surface catalyze the reaction of polymerization of tars which lead to coke formation and deposit on the catalysts, as well as generate H_2_. The formed coke could be reacted with steam to produce CO and H_2_ by steam reforming. In addition, the Me^0^ in the oxidizing atmosphere can be oxidized, giving rise to different valence states in metal oxides.

## 4. Conclusions

Ex situ catalytic pyrolysis of biomass for syngas production and tar decomposition based on char-supported Fe and Ni catalyst was investigated. Ni/char catalyst exhibited the highest catalytic performance: The syngas yield and the molar ratio of H_2_/CO were 0.91 Nm^3^/kg biomass and 1.64, respectively. Moreover, the lowest tar yield (43.21 g/kg biomass) and the highest tar catalytic conversion efficiency (84.97 wt.%) were also obtained under the condition of Fe-Ni/char. After catalytic pyrolysis, the percentage of aromatic hydrocarbons in tar dramatically increased with the significant decrease in oxygenated compounds and nitrogenous compounds, especially in the presence of Fe-Ni/char catalyst. The XPS analysis results indicated that the metallic oxides (Me_x_O_y_) were reduced to metallic Me^0^ after catalytic pyrolysis promoted the increase of tar conversion.

## Figures and Tables

**Table 1:** Ultimate and proximate analyses of pine sawdust (PS) and char samples ^b.^

	Proximate Analysis (wt.%)				Ultimate Analysis (wt.%)					S*_BET_*
	M	A	V	FC	C	H	O ^a^	N	S	(m^2^/g)
PS	9.18	1.25	74.46	15.11	46.36	5.75	45.3	2.26	0.35	5.24
Char	3.04	10.4	5.45	81.11	76.12	1.01	8.12	2.54	0.27	252.13

Notes: M, moisture content; V, volatile matter; F, fixed carbon; A, ash; LHV, low heating value; *S_BET_*, Brunauer–Emmett–Teller specific surface area. ^a^ By difference. ^b^ Air dry basis.

**Table 2:** Textural properties of fresh char and char-supported catalysts.

Sample.	BET Surface Area (S*_BET_*: m^2^/g)	Total Pore Volume (V_*total*_: cm^3^/g)	Average Pore Diameter (D: nm)
Char	252.13	0.20	3.56
Fe-Ni/char	74.89	0.06	2.42

**Table 3:** Elemental analysis of tars from different catalysts.

Catalyst	C	H	N	S	O ^a^	H/C ^b^	O/C ^b^	Molecular Formula
	wt.%	wt.%	wt.%	wt.%	wt.%			
No catalyst	54.67	7.219	6.2	0.048	31.863	1.585	0.437	CH_1.585_O_0.437_N_0.097_
Char	57.45	7.04	5.43	0.023	30.057	1.47	0.392	CH_1.471_O_0.392_N_0.097_
Fe-Ni/char	77.43	6.13	4.56	0.024	11.856	0.95	0.115	CH_0.95_O_0.114_N_0.069_

^a^ By difference; ^b^ Molar ratio.

## Supplementary Materials

The following are available online at https://www.mdpi.com/2079-4991/10/7/1397/s1. Figure S1: SEM-EDS of different fresh catalysts. (a) char, (b) Fe-Ni/char. Figure S2: Pore size distributions and nitrogen adsorption-desorption isotherms of different fresh catalysts. (a) char, (b) Fe-Ni/char. Table S1. Main compounds identified in the tar product by GC-MS analysis
