# Supplementary material for: Insight into the Ex Situ Catalytic Pyrolysis of Biomass over Char Supported Metals Catalyst: Syngas Production and Tar Decomposition
Source: Nanomaterials (Basel). 2020 Jul 18;10(7):1397. doi: 10.3390/nano10071397 (PMC7407590; doi:10.3390/nano10071397)
Supplement: Supplementary file 1 [file nanomaterials-10-01397-s001.pdf]

nanomaterials

Supplementary material

# Insight into the Ex Situ Catalytic Pyrolysis of Biomass over Char Supported Metals Catalyst: Syngas Production and Tar Decomposition

Mian Hu [1,2], Baihui Cui [3], Bo Xiao [2], Shiyi Luo [4], Dabin Guo [2,*]

[1] College of Environment, Zhejiang University of Technology, Hangzhou 310032, Zhejiang, China; mianhu@zjut.edu.cn

[2] School of Environmental Science & Engineering, Huazhong University of Science and Technology, Wuhan 430074, China; xiaobo1958@126.com

[3] Institute of Hydrobiology, Chinese Academy of Sciences, Wuhan 430072, China; cuibaihui@ihb.ac.cn

[4] State Key Laboratory of Physical Chemistry of Solid Surfaces, College of chemistry and chemical Engineering, Xiamen University, Xiamen 361005, China; luoshiyi@xmu.edu.cn

* Correspondence: dabin@hust.edu.cn

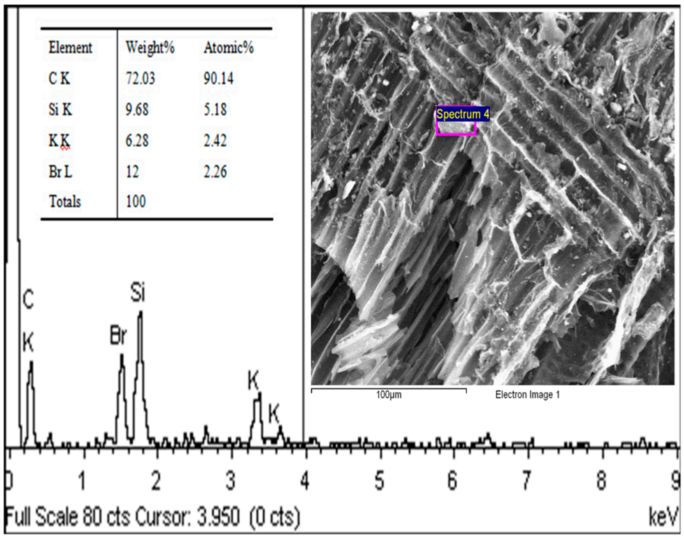


(a)

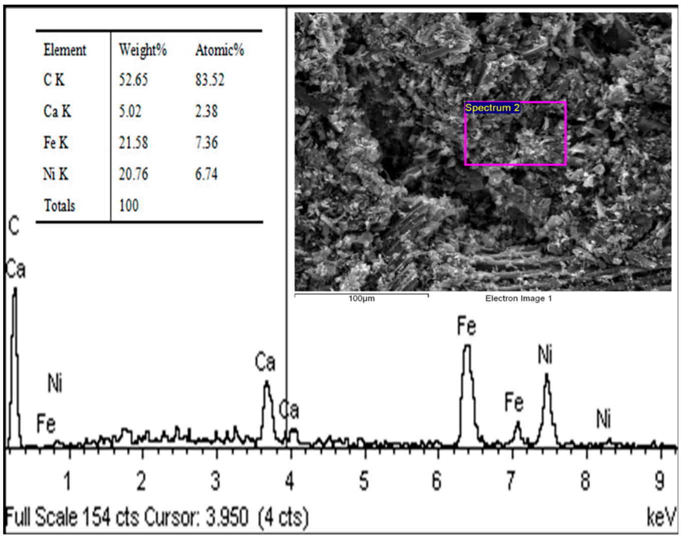


(b)

**Figure S1.** SEM-EDS of different fresh catalysts. (a) char, (b) Fe-Ni/char.

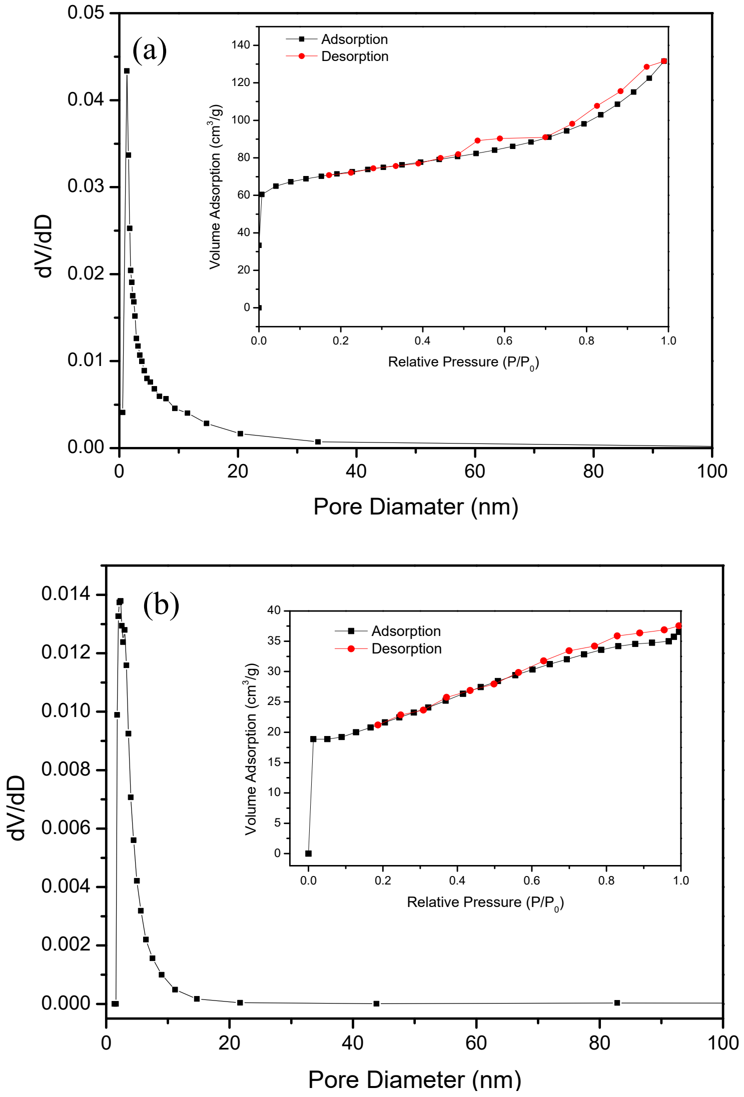


**Figure S2.** Pore size distributions and nitrogen adsorption-desorption isotherms of different fresh catalysts. (a) char, (b) Fe-Ni/char.

Table S1 Main compounds identified in the tar product by GC-MS analysis

| Compounds | Formula | Area (%) | | |
|---|---|---|---|---|
| | | no catalyst | char | Fe-Ni/char |
| **Aromatic hydrocarbons** | | | | |
| Benzene | $C_6H_6$ | 4.8579 | 11.8503 | 10.7966 |
| Toluene | $C_7H_8$ | 12.7847 | 15.1737 | 16.9579 |
| p-Xylene | $C_8H_{10}$ | 1.5401 | 1.4995 | — |
| Benzene, 1,3-dimethyl- | $C_8H_{10}$ | — | — | 1.5868 |
| Styrene | $C_8H_8$ | 2.6404 | 3.4748 | 3.6313 |
| Phenylethyne | $C_8H_6$ | — | 0.241 | — |
| Benzene, (1-methylethyl)- | $C_9H_{12}$ | 0.0527 | — | — |
| Benzene, 1-ethyl-3-methyl- | $C_9H_{12}$ | 0.373 | 0.2024 | 0.1959 |
| Benzene, 1-ethyl-2-methyl- | $C_9H_{12}$ | 0.1267 | — | — |
| Mesitylene | $C_9H_{12}$ | — | 0.1041 | 0.1286 |
| Benzene, 1,2,4-trimethyl- | $C_9H_{12}$ | — | — | 0.0699 |
| Benzene, 1,2,3-trimethyl- | $C_9H_{12}$ | — | — | — |
| Benzene, 1-ethenyl-3-methyl- | $C_9H_{10}$ | 0.1059 | — | — |
| Benzene, 2-propenyl- | $C_9H_{10}$ | 0.0905 | — | 0.1105 |
| Benzene, 1-propenyl- | $C_9H_{10}$ | — | 0.0775 | — |
| Benzene, cyclopropyl- | $C_9H_{10}$ | — | 0.0563 | — |
| Indane | $C_9H_{10}$ | — | | — |
| Indene | $C_9H_8$ | 1.3976 | 3.137 | 2.7677 |
| 2-Methylindene | $C_{10}H_{10}$ | 1.8195 | 0.4379 | 0.4744 |
| Cycloprop[a]indene, 1,1a,6,6a-tetrahydro- | $C_{10}H_{10}$ | — | 0.3835 | — |
| 1,4-Dihydronaphthalene | $C_{10}H_{10}$ | — | 0.1175 | 0.1114 |
| Benzene, 1,3-diethenyl- | $C_{10}H_{10}$ | — | — | — |
| Benzene, (1-methyl-2-cyclopropen-1-yl)- | $C_{10}H_{10}$ | — | — | 0.4105 |
| Naphthalene | $C_{10}H_8$ | 1.7578 | 5.0898 | 4.4736 |
| Naphthalene, 1-methyl- | $C_{11}H_{10}$ | — | 0.1195 | — |
| Naphthalene, 2-methyl- | $C_{11}H_{10}$ | 1.3828 | 1.893 | 1.6251 |
| Biphenyl | $C_{12}H_{10}$ | 0.5894 | 0.3456 | 0.2911 |
| Naphthalene, 1-ethyl- | $C_{12}H_{12}$ | 0.1813 | — | — |
| Naphthalene, 2-ethyl- | $C_{12}H_{12}$ | 0.1423 | — | — |
| Naphthalene, 1,3-dimethyl- | $C_{12}H_{12}$ | 0.1997 | 0.0664 | — |
| Naphthalene, 2,3-dimethyl- | $C_{12}H_{12}$ | — | — | — |
| Naphthalene, 1,4-dimethyl- | $C_{12}H_{12}$ | 0.1488 | 0.1571 | — |

| | | | | |
|---|---|---|---|---|
| Naphthalene, 1,6-dimethyl- | $C_{12}H_{12}$ | 0.1458 | 0.0814 | — |
| Naphthalene, 2,6-dimethyl- | $C_{12}H_{12}$ | — | — | 0.0819 |
| Naphthalene, 2,7-dimethyl- | $C_{12}H_{12}$ | — | — | 0.137 |
| Naphthalene, 2-ethenyl- | $C_{12}H_{10}$ | 0.4193 | 0.3221 | 0.2628 |
| Acenaphthene | $C_{12}H_{10}$ | — | 0.1608 | 0.1302 |
| Biphenylene | $C_{12}H_{8}$ | 0.5303 | 1.0857 | 0.8799 |
| Naphthalene, 2-(1-methylethenyl)- | $C_{13}H_{12}$ | 0.4072 | — | — |
| Fluorene | $C_{13}H_{10}$ | 0.4339 | 0.9376 | 0.5974 |
| 3H-Benz[e]indene, 2-methyl- | $C_{14}H_{12}$ | 0.1077 | — | — |
| 1,1'-Biphenyl, 4-ethenyl- | $C_{14}H_{12}$ | 0.1758 | 0.0348 | — |
| 9H-Fluorene, 2-methyl- | $C_{14}H_{12}$ | — | 0.1177 | — |
| 9H-Fluorene, 1-methyl- | $C_{14}H_{12}$ | — | 0.068 | — |
| Anthracene | $C_{14}H_{10}$ | 1.3293 | 0.7347 | 0.8525 |
| Phenanthrene | $C_{14}H_{10}$ | — | 0.2944 | — |
| Dibenzo[a,e]cyclooctene | $C_{16}H_{12}$ | 0.1227 | — | — |
| Anthracene, 2-methyl- | $C_{15}H_{12}$ | 0.0659 | — | — |
| Anthracene, 1-methyl- | $C_{15}H_{12}$ | 0.0634 | — | — |
| Phenanthrene, 2-methyl- | $C_{15}H_{12}$ | 0.1324 | — | 0.1877 |
| Phenanthrene, 1-methyl- | $C_{15}H_{12}$ | 0.1002 | 0.3682 | 0.1028 |
| Naphthalene, 2-phenyl- | $C_{16}H_{12}$ | 0.2833 | — | — |
| Fluoranthene | $C_{16}H_{10}$ | 0.4638 | 0.1406 | 0.1817 |
| Pyrene | $C_{16}H_{10}$ | — | 0.2894 | 0.2272 |
| p-Terphenyl | $C_{18}H_{14}$ | 0.1937 | — | — |
| m-Terphenyl | $C_{18}H_{14}$ | 0.1465 | — | — |
| Pyrene, 1-methyl- | $C_{17}H_{12}$ | 0.1038 | 0.1108 | — |
| Triphenylene | $C_{18}H_{12}$ | 0.1152 | — | — |
| $\sum$ | | **35.5313** | **49.173** | **47.2724** |
| **Oxygenated compounds** | | | | |
| Methyl propionate | $C_4H_8O_2$ | 2.2663 | — | — |
| Ethyl Acetate | $C_4H_8O_2$ | — | 2.2288 | 1.8793 |
| 2-Cyclopenten-1-one | $C_5H_6O$ | 0.7669 | — | — |
| Propanoic acid, 2-methyl-, methyl ester | $C_5H_{10}O_2$ | — | — | — |
| Phenol | $C_6H_6O$ | 3.1039 | 2.534 | 2.4583 |
| 2-Cyclopenten-1-one, 2-methyl- | $C_6H_8O$ | 0.2995 | — | — |
| 2-Cyclopenten-1-one, 3-methyl- | $C_6H_8O$ | 0.3943 | — | — |
| Phenol, 2-methyl- | $C_7H_8O$ | 0.9959 | 0.4063 | 0.3739 |
| p-Cresol | $C_7H_8O$ | — | — | — |
| Acetic acid, phenyl ester | $C_8H_8O_2$ | — | — | — |

| | | | | |
|---|---|---|---|---|
| Acetophenone | $C_8H_8O$ | 0.2356 | — | — |
| Phenol, 3-methyl- | $C_7H_8O$ | 2.5113 | 0.7561 | 0.4318 |
| Phenol, 2,3-dimethyl- | $C_8H_{10}O$ | 0.1927 | — | — |
| Phenol, 2,4-dimethyl- | $C_8H_{10}O$ | 0.1386 | — | — |
| Cinnamaldehyde, (E)- | $C_9H_8O$ | — | — | — |
| Butylated Hydroxytoluene | $C_{15}H_{24}O$ | — | — | — |
| Diisooctyl phthalate | $C_{24}H_{38}O_4$ | 0.0465 | — | — |
| 1,3-Dioxolane, 2-methyl- | $C_4H_8O_2$ | 4.1942 | 4.6633 | 0.0752 |
| Furan, 2,5-dimethyl- | $C_6H_8O$ | 0.1772 | — | — |
| Benzofuran | $C_8H_6O$ | 1.6827 | 2.0057 | 2.1845 |
| Benzofuran, 7-methyl- | $C_9H_8O$ | — | 0.5901 | — |
| Benzofuran, 2-methyl- | $C_9H_8O$ | 0.2876 | — | 0.5747 |
| ∑ | | **17.2932** | **13.1843** | **7.9777** |
| **Nitrogenous compounds** | | | | |
| 1,3-Diazine | $C_4H_4N_2$ | 0.1561 | — | — |
| Pyrazine | $C_4H_4N_2$ | — | 0.1096 | 0.1113 |
| Pyridine | $C_5H_5N$ | 2.9669 | — | — |
| Pyrrole | $C_4H_5N$ | 1.2094 | 1.3204 | 1.214 |
| Pyridine, 2-methyl- | $C_6H_7N$ | 0.5314 | 0.4597 | 0.4687 |
| Pyrazine, methyl- | $C_5H_6N_2$ | 0.4105 | — | — |
| 1H-Pyrrole, 3-methyl- | $C_5H_7N$ | — | 0.0924 | — |
| 1H-Pyrrole, 2-methyl- | $C_5H_7N$ | 0.0984 | — | — |
| Pyridine, 2-ethenyl- | $C_7H_7N$ | — | 0.0546 | — |
| Pyridine, 2,5-dimethyl- | $C_7H_9N$ | 0.1668 | 0.0566 | — |
| Pyridine, 3,5-dimethyl- | $C_7H_9N$ | — | — | 0.0617 |
| Benzyl nitrile | $C_8H_7N$ | — | 0.1281 | 0.11 |
| Benzonitrile, 2-methyl- | $C_8H_7N$ | — | 0.1267 | — |
| Quinoline | $C_9H_7N$ | 0.255 | — | — |
| 4-Piperidinone, 2,2,6,6-tetramethyl- | $C_9H_{17}NO$ | — | 0.2461 | — |
| Isoquinoline | $C_9H_7N$ | — | 0.0765 | — |
| ∑ | | **5.7945** | **2.6707** | **1.9657** |
